# A mitochondrion-associated PPR protein, WBG1, regulates grain chalkiness in rice

**DOI:** 10.3389/fpls.2023.1136849

**Published:** 2023-03-09

**Authors:** Mingming Wu, Maohong Cai, Rongrong Zhai, Jing Ye, Guofu Zhu, Faming Yu, Shenghai Ye, Xiaoming Zhang

**Affiliations:** ^1^ Institute of Crop and Nuclear Technology Utilization, Zhejiang Academy of Agricultural Sciences, Hangzhou, China; ^2^ College of Life and Environmental Science, Hangzhou Normal University, Hangzhou, China

**Keywords:** grain chalkiness, rice, *wbg1*, white belly, mitochondrion, grain width

## Abstract

Rice kernel quality has vital commercial value. Grain chalkiness deteriorates rice’s appearance and palatability. However, the molecular mechanisms that govern grain chalkiness remain unclear and may be regulated by many factors. In this study, we identified a stable hereditary mutant, *white belly grain 1* (*wbg1*), which has a white belly in its mature grains. The grain filling rate of *wbg1* was lower than that of the wild type across the whole filling period, and the starch granules in the chalky part were oval or round and loosely arranged. Map-based cloning showed that *wbg1* was an allelic mutant of *FLO10*, which encodes a mitochondrion-targeted P-type pentatricopeptide repeat protein. Amino acid sequence analysis found that two PPR motifs present in the C-terminal of WBG1 were lost in wbg1. This deletion reduced the splicing efficiency of *nad1* intron 1 to approximately 50% in *wbg1*, thereby partially reducing the activity of complex I and affecting ATP production in *wbg1* grains. Furthermore, haplotype analysis showed that *WBG1* was associated with grain width between *indica* and *japonica* rice varieties. These results suggested that *WBG1* influences rice grain chalkiness and grain width by regulating the splicing efficiency of *nad1* intron 1. This deepens understanding of the molecular mechanisms governing rice grain quality and provides theoretical support for molecular breeding to improve rice quality.

## Introduction

Grain chalkiness describes the presence of opaque spots in the endosperm and has a vital role in determining rice quality. Rice grains with high transparency and low chalkiness are preferred by consumers, and varieties that produce this type of grain are popular with rice farmers and seed companies (Fitzgerald et al., 2009). However, rice varieties with high chalkiness grains are difficult to be popularized on a large scale, despite the high yield. Grain chalkiness not only affects the appearance and palatability of rice but also results in reduced yield after rice grain processing (Li et al., 2014; Misra et al., 2019). Therefore, clarification of the molecular mechanisms of rice chalkiness could contribute to the development of high-yield varieties with high quality to meet the market demand.

There are three types of rice grain chalkiness, dependent on where the chalkiness appears in the grain: white belly, white core, and white back (Zhou et al., 2009; Siebenmorgen et al., 2013). In the chalky part of the endosperm, starch granules are round and loosely packed, with many air gaps between starch granules, resulting in low light transmittance and a chalky appearance. In contrast to the chalky part, starch granules are polyhedral and densely packed in the transparent part (Guo et al., 2011; Li et al., 2014). Previous studies have shown that starch synthesis genes, storage protein biosynthesis regulation genes, and plastid and mitochondrion-associated genes may regulate chalkiness (Zhao et al., 2022).

In the starch synthesis pathway, the coordination and balance of various enzyme activities are an essential prerequisite for transparent grain formation. Weak or loss-of-function mutations of rice endosperm ADP-glucose pyrophosphorylase large subunit OsAGPL2 lead to various phenotypic mutations in grain type, ranging from white core to shrunken floury (Lee et al., 2007; Tuncel et al., 2014; Tang et al., 2016; Wei et al., 2017). The small subunit of ADP-glucose pyrophosphorylase qACE9 determines the size of the area of the chalky endosperm in rice (Gao et al., 2016). *OsBT1* encodes an ADP-glucose transporter localized in the amyloplast envelope membrane. The *osbt1* null mutant has a white core endosperm (Cakir et al., 2016; Li et al., 2017). RNA interference (RNAi)-repressed or knockout *SSIIIa* mutants had grains with white core endosperms (Ryoo et al., 2007; Zhang et al., 2011). Mutation of *SSIIa via* RNAi repression slightly increased grain chalkiness area, which resulted in a little white belly or white back. The kernels of *SSIIa*/*SSIIIa* double-repression mutant rice lines also had a chalky kernel appearance (Ryoo et al., 2007; Zhang et al., 2011). *PHO1* encodes a plastidial α-glucan phosphorylase, and *pho1* mutants have a white core or shrunken endosperm, which is temperature-dependent (Satoh et al., 2008). OsbZIP58, a bZIP transcription factor, was shown to directly bind to the promoters of six starch-synthesizing genes, including *OsAGPL3*, *Wx*, *OsSSIIa*, *SBE1*, *OsBEIIb*, and *ISA2*, to regulate their expression. *Osbzip58* null mutants had white-bellied grains, with reduced starch accumulation in the grain belly region (Wang et al., 2013). RNAi repression or knockout of the nuclear transcription factor *NF-YB1* decreased the filling rate of grains and resulted in a chalky grain phenotype (Xu et al., 2016; Bello et al., 2019). Knockout of *NF-YC12* significantly reduced the accumulation of starch and increased grain chalkiness (Bello et al., 2019; Xiong et al., 2019). RSR1, an AP2/EREBP transcription factor, negatively regulates starch synthesis in the endosperm (Fu and Xue, 2010). The *rsr1* mutant had altered starch granule morphology, resulting in a white core phenotype (Fu and Xue, 2010).

In seed storage protein biosynthesis, defects in post-transcriptional processing of storage proteins will lead to a complete floury endosperm phenotype (Wang et al., 2010; Fukuda et al., 2011; Tian et al., 2013; Ren et al., 2014; Fukuda et al., 2016; Wang et al., 2016). Recent studies found that some transcription factors and unfolded protein response (UPR) genes are involved in rice seed storage protein metabolism and regulation of grain chalkiness. *OsbZIP50*, *OsBip1*, *OsBip2*, and *OsBip3* encode proteins that promote endoplasmic reticulum (ER) UPR, and rice lines that overexpress these UPR genes had various degrees of chalkiness. OsbZIP60 inhibits the expression of *OsbZIP50*, *OsBip1*, *OsBip2*, and *OsBip3* to maintain ER homeostasis, and *Osbzip60* showed high chalkiness (Yang et al., 2022b).

Defects in genes regulating plastid and mitochondrial metabolism can also lead to grain chalkiness. Mutation of the chloroplast-specific genes pyruvate orthophosphate dikinase *FLO4*, alanine aminotransferase *LNUE1*, and glyoxalase *FLO15/OsGLYI7* resulted in white core endosperm phenotypes (Kang et al., 2005; You et al., 2019; Fang et al., 2022). *OsGBP* encodes a plastid-located granule-bound starch synthase binding protein with a carbohydrate-binding module 48 (CBM48) domain; mutation of this gene yields a white belly phenotype (Wang et al., 2020). In addition, the mutation of genes regulating plastid size and differentiation in the endosperm also results in increased grain chalkiness (Matsushima et al., 2014; Cai et al., 2018). The mitochondrion-associated pyruvate kinase OsPK3 physically forms heterodimers with two other PK isozymes, OsPK1 and OsPK4, which are involved in the regulation of grain filling; loss-of-function mutations in *OsPK1*, *OsPK3*, or *OsPK4* led to chalky grains (Hu et al., 2020).

Previous studies have revealed that deficiency in mitochondrial energy supply leads to serious grain filling defects, with a floury rice grain phenotype (Hao et al., 2019; Wu et al., 2019; Yu et al., 2021). However, it remains unclear whether PPR protein and mitochondrial energy supply defects affect grain chalkiness. In this study, we addressed this question by generating a stable hereditary mutant, *wbg1*, which yielded a white belly phenotype. Our results confirmed that *WBG1* encodes a mitochondrion-targeted P-type PPR protein and affects rice grain chalkiness by regulating the splicing efficiency of *nad1* intron 1.

## Materials and methods

### Plant material and growth conditions

The *wbg1* mutant was identified from a mutant pool created by treating the *indica* cultivar N22 seeds with *N*-methyl-*N*-nitrosourea (MNU). *flo10* is a rice floury grain mutant that was identified in our previous study (Wu et al., 2019). Developing seeds and mature seeds were taken from the plants growing in paddy fields during the normal growing season.

### Microscopy

Scanning electron microscopy (SEM) was used to observe grain phenotype. For this, mature brown grains of wild-type and *wbg1* plants were transversely cut using a razor blade and sputter-coated with gold, followed by observation using a HITACHI S-3000N scanning electron microscope. For semi-thin section observation, a transverse section of developing endosperms approximately 1 mm thick was fixed in 2.5% (v/v) glutaraldehyde and 1% (w/v) paraformaldehyde. Samples were sectioned and observed as described previously (Wu et al., 2019).

### Positional cloning, vector construction, and rice transformation

For *WBG1* mapping, more than 120 polymorphic simple sequence repeat (SSR) and insertion–deletion (InDel) markers covering the whole rice genome were used. Molecular markers in the candidate regions were designed based on nucleotide polymorphisms identified between the *indica* cultivar 9311 and the *japonica* cultivar Nipponbare (https://ensembl.gramene.org/Oryza_indica/Location/Compara_Alignments). The *WBG1* locus was narrowed to a 98.9-kb region on rice chromosome 3 using the primers listed in **Supplementary Table 1**. Annotations of predicted open reading frames (ORFs) were determined using genomic sequence NC_029258.1 (3639936 to 3738852) from the National Center for Biotechnology Information (https://www.ncbi.nlm.nih.gov/nuccore/NC_029258.1).

For genetic complementation tests, a 6.1-kb fragment containing the promoter and the full-length coding sequence of *WBG1* was amplified using the primers listed in **Supplementary Table 1**. The fragment was cloned into the binary plasmid vector *pCAMBIA1390*, followed by introduction into *Agrobacterium tumefaciens* strain EHA105 and infection of the calli of *wbg1*. Positive transgenic lines were selected using the recombined vector-specific primers listed in **Supplementary Table 1**.

### Blue native polyacrylamide gel electrophoresis and activity staining of NADH dehydrogenase

Blue native polyacrylamide gel electrophoresis (BN-PAGE) and activity staining of NADH dehydrogenase were performed as previously described (Wu et al., 2019).

### RNA extraction, reverse transcriptase polymerase chain reaction, and quantitative RT-PCR analysis

RNA from grains at 9 days after fertilization (DAF) was extracted using an RNAprep Pure Plant Kit (Tiangen Co., Beijing, China) and reverse-transcribed using PrimeScript™ II Reverse Transcriptase (TaKaRa, Mountain View, CA, USA) with random hexamer primers. Reverse transcriptase polymerase chain reaction (RT-PCR) analyses of each splicing event in *nad1* introns were performed using primers as previously described (Wu et al., 2019). The rice *Actin* gene was used as an internal control.

qRT-PCR analyses were performed using an ABI 7500 real-time PCR system with the SYBR Premix Ex Taq™ Kit (TaKaRa). qRT-PCR to assess the splicing efficiency of *nad1* intron 1 and *nad1* exon1 precursors was performed using the primers listed in **Supplementary Table 1**. The rice *Ubiquitin* gene was used as an internal control.

### Measurement of total starch, amylose, and ATP content

The total starch content of wild-type and *wbg1* mature grains was measured using a Total Starch Assay Kit (Megazyme, Bray, Ireland). The amylose content of wild-type and *wbg1* mature grain was measured using the concanavalin A (Con A)-based method, described previously (Wu et al., 2019). ATP content of wild-type and *wbg1* grains at 9 DAF were measured using a luciferin–luciferase ATP assay kit following the manufacturer’s protocol (Beyotime, Jiangsu, China).

### Measurement of DNA density in agarose gel electrophoresis

Each lane of the gel was plotted using ImageJ software to obtain the value curve (https://imagej.net/software/imagej/). The areas under each resultant value curve were measured in pixels using the wand (tracing) tool in ImageJ.

### Haplotypes analysis of *WBG1*

To analyze the haplotypes of *WBG1* in controlling grain width, the full-length chromosome region of *WBG1* (3,914 bp, Chr3: 3690005 to 3693919) was used to BLAST in RFGB database (https://www.rmbreeding.cn/). The resources of 1,649 *indica* and 795 *japonica* cultivars and the data of grain width (GW) were all collected from the RFGB database. The statistical analysis of phenotypic differences between haplotypes was obtained by using a methodology based on the one-way ANOVA-protected Tukey’s *t*-tests multiple pairwise comparisons indicated in https://www.rmbreeding.cn/Index/manual#haplotype.

### Statistical analysis

GraphPad Prism 8.0 (http://www.graphpad.com/) was used for the statistical analysis. Student’s *t*-test was used to examine the experimental data.

## Results

### Phenotypic characterization of the *wbg1* mutant

**Figure 1:**
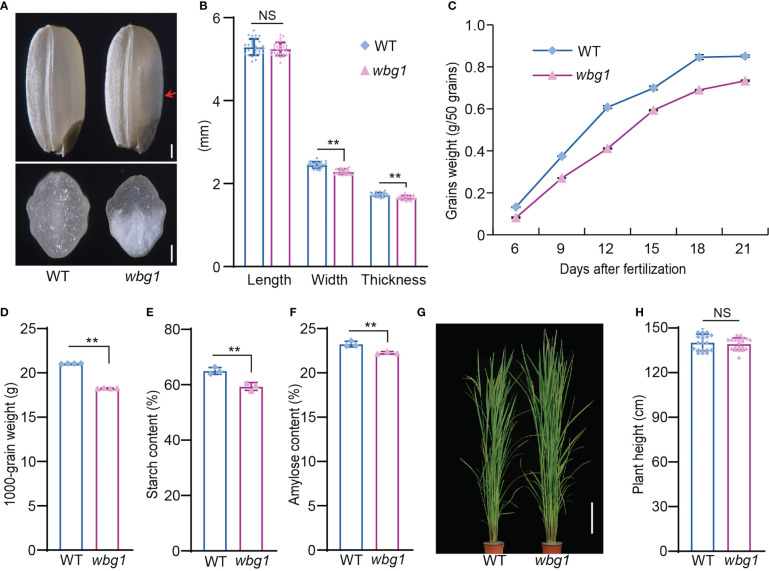
*wbg1* phenotype. **(A)** The outer (upper panel) and cross-section (lower panel) phenotypes of wild-type (WT) and *wbg1* grains. The belly side of the *wbg1* grain is indicated by the red arrowhead. **(B)** Grain length, width, and thickness of WT and *wbg1* grains. **(C)** Grain filling rates of WT and *wbg1*. **(D)** Thousand-grain weights of WT and *wbg1*. **(E)** Starch content of WT and *wbg1* grains. **(F)** Amylose content of WT and *wbg1* grains. **(G)** Plant phenotypes of WT and *wbg1* at the grain-filling stage. Scale bar = 20 cm. **(H)** Plant heights of WT and *wbg1* at the grain filling stage. Values are presented as mean ± standard deviation (SD) (**p < 0.01; Student’s *t*-test). NS, not significant.

Rice grain chalkiness leads to reductions in rice grain quality and yield. To study the mechanism governing rice grain chalkiness, we treated the *indica* cultivar N22 grains with MNU and screened mutants for decreased grain quality and weight. Among them, a mutant with white belly phenotype was isolated and named *white belly grain 1* (*wbg1*) (**Figure 1A**). Compared with the wild type, the grain length of *wbg1* was unchanged, but grain width and thickness were significantly decreased (**Figure 1B**). The filling rate of *wbg1* grains across the whole filling period was lower than that in the wild type, and the thousand-grain weight of *wbg1* mature seeds was also decreased (**Figures 1C, D**). Starch measurement showed that the total starch and amylose contents were reduced in the *wbg1* mutant (**Figures 1E, F**). Although *wbg1* had decreased grain quality and weight, there was no significant difference in plant height when compared with the wild type (**Figures 1G, H**). Together, these results indicated that the mutation in *wbg1* led to a decrease in grain filling rate and a white belly phenotype in mature grains.

### Starch granules in the belly side of *wbg1* grains were loosely packed

**Figure 2:**
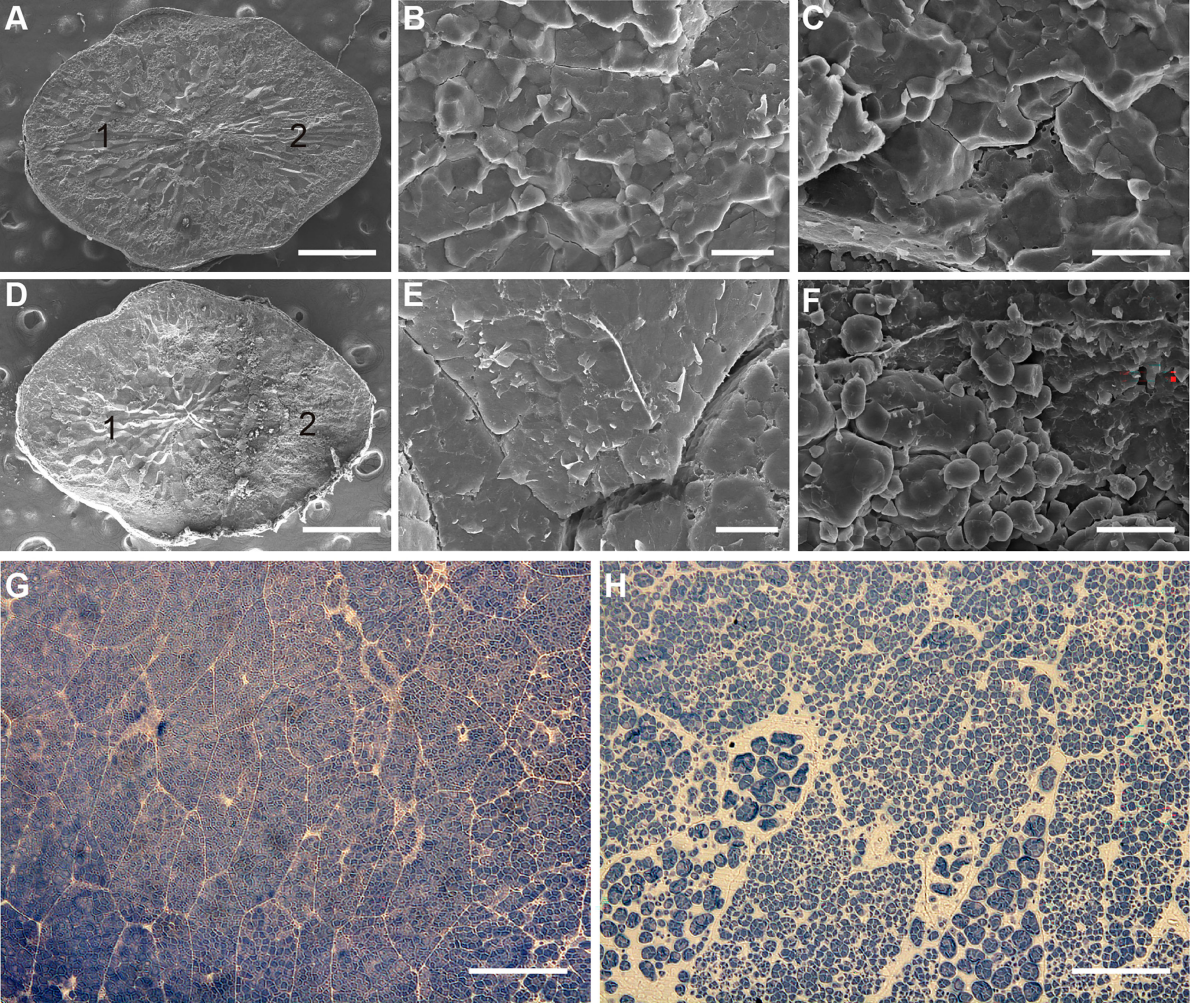
*wbg1* endosperm morphology. **(A–F)** Scanning electron micrographs of transverse sections of wild-type (WT) **(A–C)** and *wbg1* **(D–F)** endosperm. **(A, D)** Intact endosperm. “1” and “2” indicate the dorsal and belly sides of the endosperm, respectively. Scale bars, 0.5 mm. **(B, E)** Magnified region of the dorsal side of WT and *wbg1* endosperm, respectively. Scale bars, 10 μm. **(C, F)** Magnified region of the belly side of WT and *wbg1* endosperm, respectively. Scale bars, 10 μm. **(G, H)** Semi-thin sections of the belly side of WT and *wbg1* endosperm, respectively. Scale bars, 10 μm.

To investigate the mechanism of white belly formation in *wbg1*, scanning electron microscopy was performed to examine the belly and dorsal sides of *wbg1* grains. The arrangement of starch granules on the dorsal side of *wbg1* grains was consistent with that of the wild type, where starch granules were polyhedral and tightly packed. However, starch granules in the belly side of *wbg1* grains were mostly oval or round and loosely arranged (**Figures 2A–F**). Semi-thin sections were cut to assess compound starch grains in the belly side of the developing endosperm in *wbg1*. The results showed that these starch grains were not tightly arranged and were fragile (**Figures 2G, H**). These results suggested that the *wbg1* mutation resulted in the loose arrangement of starch granules in the belly side of the endosperm, which led to the white belly phenotype.

### *wbg1* is a weak allelic mutant of *FLO10*

**Figure 3:**
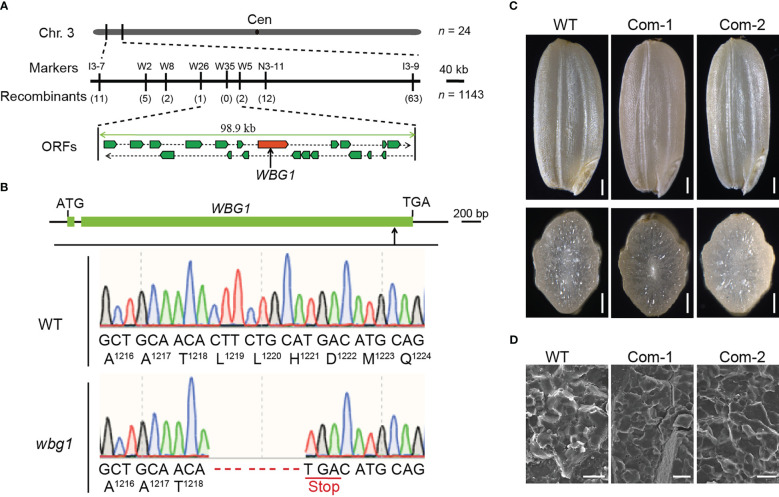
*WBG1* map-based cloning and complementation tests. **(A)** Fine mapping of the *WBG1* locus. The *WBG1* locus was narrowed to a 98.9-kb region on rice chromosome 3 by using fine mapping primers listed in **Supplementary Table 1**. This region includes 20 predicted open reading frames (ORFs). The molecular markers and numbers of recombinants are indicated. Cen, centromere. **(B)** Exon/intron structure of *WBG1* and the mutation site in *wbg1*. Green boxes indicate the two exons, and the line between the exons indicates the intron in *WBG1*. ATG and TGA indicate the start and stop codons, respectively. An eight-nucleotide deletion in the coding region of *WBG1* results in a premature termination codon and protein truncation in *wbg1*. **(C)** Complementation of *wbg1* by the introduction of a 6.1-kb fragment containing the 2.3-kb promoter and 3.8-kb full-length coding sequence of *FLO10* into the *wbg1* mutant. Scale bar, 0.5 mm. **(D)** Scanning electron microscopy (SEM) images of the belly side of grains from representative complemented lines. Scale bar, 10 μm.

Of the 336 grains harvested from the heterozygous *WBG1*/*wbg1* plant, 265 grains showed the wild-type phenotype, 71 grains showed the *wbg1* phenotype, and the segregation ratio was consistent with 3:1 (*χ* ^2^ = 3.02 < 3.84, *p* > 0.05), indicating that *wbg1* phenotype is controlled by a single recessive nuclear gene. To identify the candidate gene responsible for the *wbg1* phenotype, map-based cloning was carried out to isolate *WBG1*. An F_2_ mapping population was created by crossing *wbg1* with the *japonica* rice cultivar Nipponbare. The mutation site in *wbg1* was initially mapped to a 1,000-kb interval between the InDel markers I3-7 and I3-9 on chromosome 3. With the use of 1,143 individuals with the *wbg1* phenotype, the *WBG1* locus was narrowed down to a 98.9-kb genomic region containing 20 ORFs (**Figure 3A**). Sequencing analysis revealed that an 8-bp deletion was detected in the 10th ORF (**Figure 3B**), previously identified as *FLO10* (Wu et al., 2019). The 8-bp deletion in *wbg1* occurred 3,651-bp downstream of the start codon, resulting in a premature stop codon that generated a truncated protein harboring the N-terminal 1,218 amino acids.

To confirm whether the weak mutation in *FLO10* was responsible for the *wbg1* mutant phenotype, we introduced a 6.1-kb fragment containing the promoter region and the full-length coding sequence of *FLO10* into the *wbg1* mutant. Both the brown rice morphology and starch grain arrangement in *wbg1* were restored (**Figures 3C, D**). Collectively, these results showed that a weak allelic mutation in *FLO10* was responsible for the *wbg1* white belly phenotype.

### Splicing efficiency of mitochondrial *nad1* intron 1 was decreased in *wbg1*

**Figure 4:**
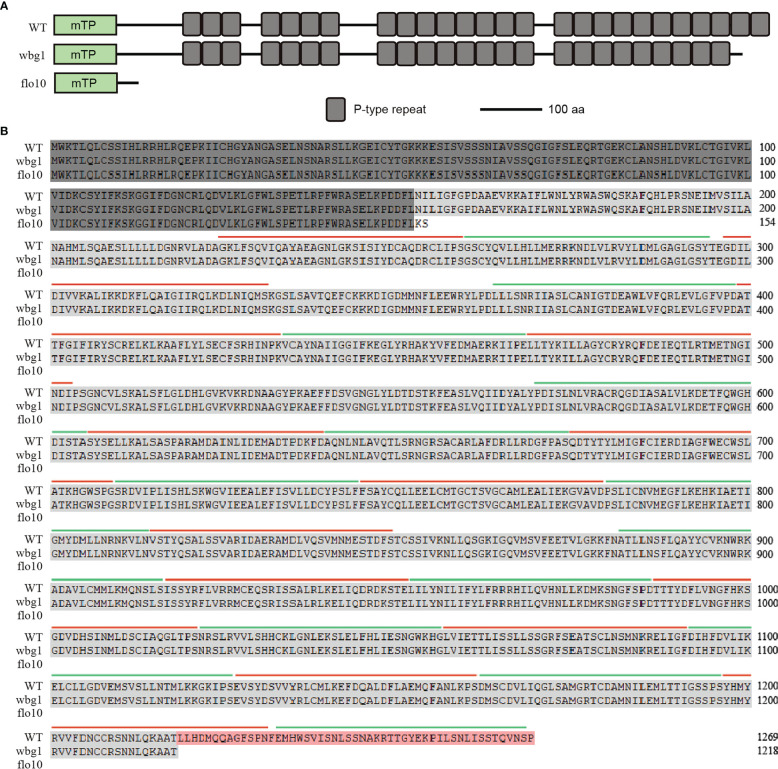
Schematic domain structure and amino acid sequence analysis of *wbg1*. **(A)** Schematic domain structure of WBG1, flo10, and wbg1 proteins. mTP, mitochondrion target peptide predicted by TargetP. WBG1/FLO10 comprises 1,269 amino acids with 26 predicted PPR repeats; the flo10 mutant lost all PPR motifs, while wbg1 only lost the last two PPR motifs at the C-terminal. **(B)** Sequences alignment of WBG1, wbg1, and flo10 proteins with predicted PPR repeat annotations. Amino acid residues present in all three sequences are indicated by dark gray shading. Light gray shading indicates the residual amino acids present in wbg1 and WBG1. The amino acid sequence at the C-terminal that is deleted in wbg1 but present in WBG1 is shaded orange. Each PPR repeat is indicated alternately by green and red lines.

**Figure 5:**
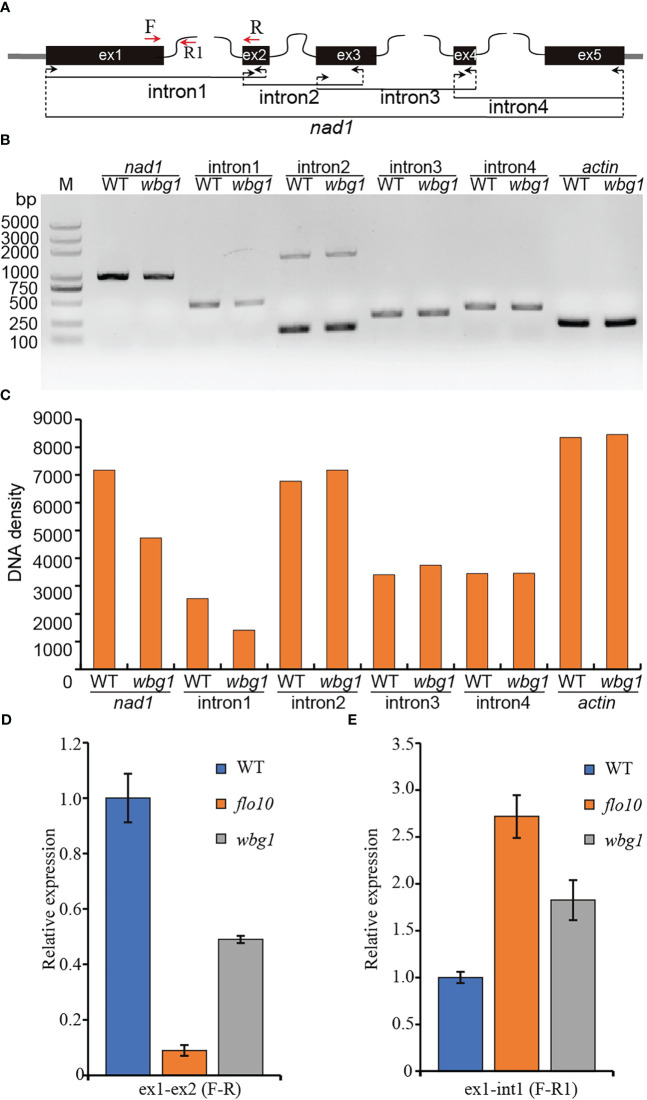
Splicing efficiency analysis of *nad1* intron 1 in *wbg1*. **(A)** Schematic representation of the rice *nad1* gene. **(B)** Reverse transcription polymerase chain reaction (RT-PCR) analysis of *nad1* intron 1, 2, 3, and 4 splicing events in wild type (WT) and *wbg1*. The amplified fragments are indicated in panel **(A)** The rice *Actin* gene was used as an internal control. **(C)** DNA density of the amplified fragments in **(B)** was quantified using ImageJ software. **(D, E)** Quantitative RT-PCR measuring the splicing efficiency of *nad1* intron 1 and *nad1* exon 1 precursor. The PCRs were performed with the primer pairs indicated in panel **(A)** RNA was extracted from WT and *wbg1* grains at 9 days after flowering (DAF). Representative results from three biological replicates are shown. Values are all mean ± standard deviation (SD).

The *flo10* mutant produces flo10 with only the N-terminal 151-amino-acid sequences and loss of all the predicted PPR motifs (Wu et al., 2019). However, the *wbg1* mutant has 1218 amino acid residues with loss of the C-terminal 51 amino acid sequences containing two PPR motifs (**Figure 4**). In the *flo10* mutant, the splicing efficiency of *nad1* intron 1 was found to be substantially decreased, with a substantial accumulation of the precursor (Wu et al., 2019). To determine whether the splicing efficiency of *nad1* intron 1 in *wbg1* mutants was affected, we carried out RT-PCR and qRT-PCR experiments. The results of RT-PCR showed that the contents of *nad1* intron 1 and mature *nad1* mRNA *in wbg1* decreased to approximately 50% (**Figures 5A–C**), which were further confirmed by qRT-PCR experiments (**Figure 5D**). The qRT-PCR experiments also indicated that *nad1* exon 1 precursor was accumulated in the *wbg1* mutant (**Figure 5E**). These results indicate that the two PPR motifs in the C-terminal, which are not present in wbg1, have an essential role in the WBG1 function.

### The *wbg1* mutant exhibits reduced respiratory chain complex I activity and ATP contents

**Figure 6:**
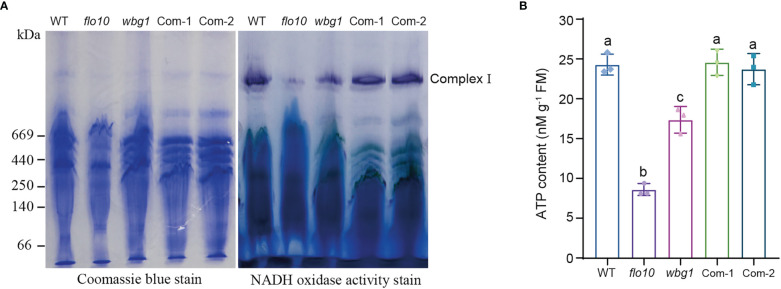
The deletion of two PPR motifs at the C-terminal of wbg1 reduced the activity of respiratory chain complex I in mitochondria. **(A)** Assembly and activity analysis of respiratory chain complex I in wild-type (WT) and *flo10* and *wbg1* mutants and complemented lines (Com-1 and Com-2). Left, Coomassie brilliant blue staining of mitochondrial respiratory chain complex I, separated using blue native gel. Right, in-gel staining of NADH dehydrogenase activity in respiratory chain complex (I) **(B)** ATP concentration in grains at 9 days after fertilization. Values are all mean ± SD from three biological replicates. a–c indicate significant differences by Student’s *t*-tests (*p* < 0.01).

*nad1* (*NADH dehydrogenase subunit 1*) encodes the core subunit of respiratory chain complex I, and the reduced splicing efficiency of *nad1* intron 1 leads to decreased *nad1*-translated protein content, which may affect the activity of respiratory chain complex I (Subrahmanian et al., 2016). BN-PAGE and in-gel NADH dehydrogenase activity staining were employed to investigate the changes in respiratory chain complex I activity in *wbg1* mutant. Compared with that in the wild type, the activity of complex I in the *wbg1* mutant was significantly reduced, but not to the extent observed in *flo10* (**Figure 6A**). The transformation of *WBG1* into the *wbg1* mutant was able to restore the activity of respiratory chain complex I to wild-type levels. Given that ATP production in mitochondria is coupled with electron transfer *via* respiratory chain complex I in mitochondria, we measured ATP content in the developing endosperm of *wbg1* and its complementary lines. The results showed that the ATP content in *wbg1* was lower than that in the wild type but higher than in the *flo10* mutant (**Figure 6B**). Transferring *WBG1* into the *wbg1* mutant was able to restore the ATP content in the developing endosperm to wild-type levels. Together, these results indicate that the grain chalkiness phenotype in *wbg1* is caused by the reduction of mitochondrial respiratory chain complex I activity resulting from the deletion of the two PPR motifs.

### *WBG1* is associated with grain width

**Figure 7:**
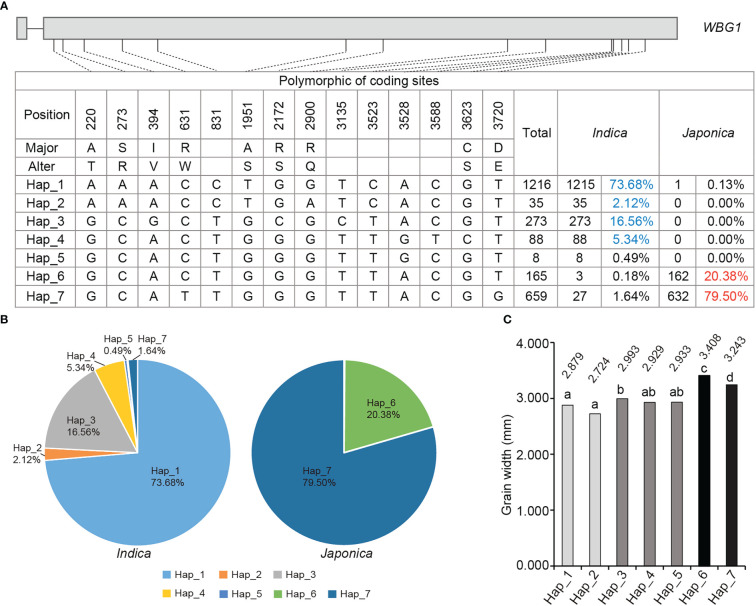
Haplotype analysis of *WBG1* and haplotype association with rice grain width. **(A)** Polymorphic nucleotides in the *WBG1* encoding region in *indica* and *japonica* cultivars. **(B)** Distribution of the proportion of seven haplotypes (Hap_1 to Hap_7) in *indica* (left) and *japonica* (right) cultivars. **(C)** Grain width statistics of the seven haplotypes. a–d indicate significant differences by Tukey’s *t*-tests (*p* < 0.01).

The *wbg1* mutant had a significantly decreased grain width (**Figure 1B**), which may result from the arrested transport of assimilates from the dorsal to belly regions of the grain. To investigate the role of *WBG1* in rice grain width, a genetic association analysis between *WBG1* and grain width was performed in *indica* and *japonica* cultivars (https://www.rmbreeding.cn). The results showed that a total of 14 polymorphic sites were found, nine of which led to amino acid changes (**Figure 7A**). Based on the polymorphic sites, seven main haplotypes (referred to as Hap_1 to Hap_7) were observed in detail in both *indica* and *japonica*. A comparison of haplotype frequencies between cultivars indicated that Hap_6 and Hap_7 were common in *japonica* cultivars, with frequencies of 20.38% and 79.50%, respectively. Hap_1 to Hap_5 were almost exclusively found in *indica* cultivars with frequencies of 73.68%, 2.12%, 16.56%, 5.34%, and 0.49%, respectively (**Figure 7B**). Hap_6 and Hap_7, the two common haplotypes in *japonica*, had wider grains (3.408 and 3.243 mm, respectively), compared with Hap_1 to Hap_5 in *indica*, which had grain widths of 2.879, 2.724, 2.993, 2.929, and 2.933 mm, respectively (**Figure 7C**). Together, these data suggest that there was clear haplotype differentiation of *WBG1* between *indica* and *japonica* rice varieties based on grain width.

## Discussion

Rice chalkiness is regulated by many genes, including those involved in starch synthesis and storage protein biosynthesis regulation, and plastid- and mitochondrion-associated genes (Zhao et al., 2022). PPR proteins form a large protein family in land plants and have functions in various processes, including cytoplasmic sterility, leaf and seed development, and stress responses (Li et al., 2021; Wang et al., 2021). The loss of function of some mitochondrion-associated PPR is known to lead to a substantial decline in grain filling rate and a floury endosperm (Hao et al., 2019; Wu et al., 2019; Yu et al., 2021; Yang et al., 2022a). However, the involvement of PPR proteins in the regulation of rice grain chalkiness has not been reported until now. In this study, we identified a stable hereditary mutant, *wbg1*, with mature grains that have a white belly phenotype (**Figure 1A**). The filling rate of *wbg1* grains across the whole filling period was lower than that of the wild type, and the starch granules in the chalky part were loosely arranged (**Figures 1C**, **2F**). Map-based cloning showed that *wbg1* was a weak allelic mutant of *FLO10* (**Figure 3**). The white belly observed in the *wbg1* mutant is different from the floury endosperm resulting from previously identified defects in mitochondrion-associated PPR proteins. Our results indicate that mitochondrion-associated PPR proteins play an important role in the rice grain white belly phenotype.

*wbg1* is a weak allele mutant of *FLO10*. In the *wbg1* mutant, there were 8-bp deletions at the tail of *WBG1*, causing a premature translation and 51-amino-acid deletion at the C-terminal (**Figure 3B**). Different from the deletion of all 26 PPR motifs in the flo10 protein, the wbg1 only lost two PPR motifs at the C-terminal (**Figure 4**). Based on the recognition code of PPR motifs (Barkan et al., 2012; Yagi et al., 2013), the last two PPR motifs may recognize two RNA bases on the *nad1* intron 1 precursor. Although the splicing efficiency of *nad1* intron 1 in *wbg1* is not as serious as that in *flo10*, it is reduced to approximately 50% (**Figure 5**). Thus, the recognition of the last two RNA bases on the *nad1* intron 1 precursor by *WBG1* plays an important role in *nad1* intron 1 splicing.

**Figure 8:**
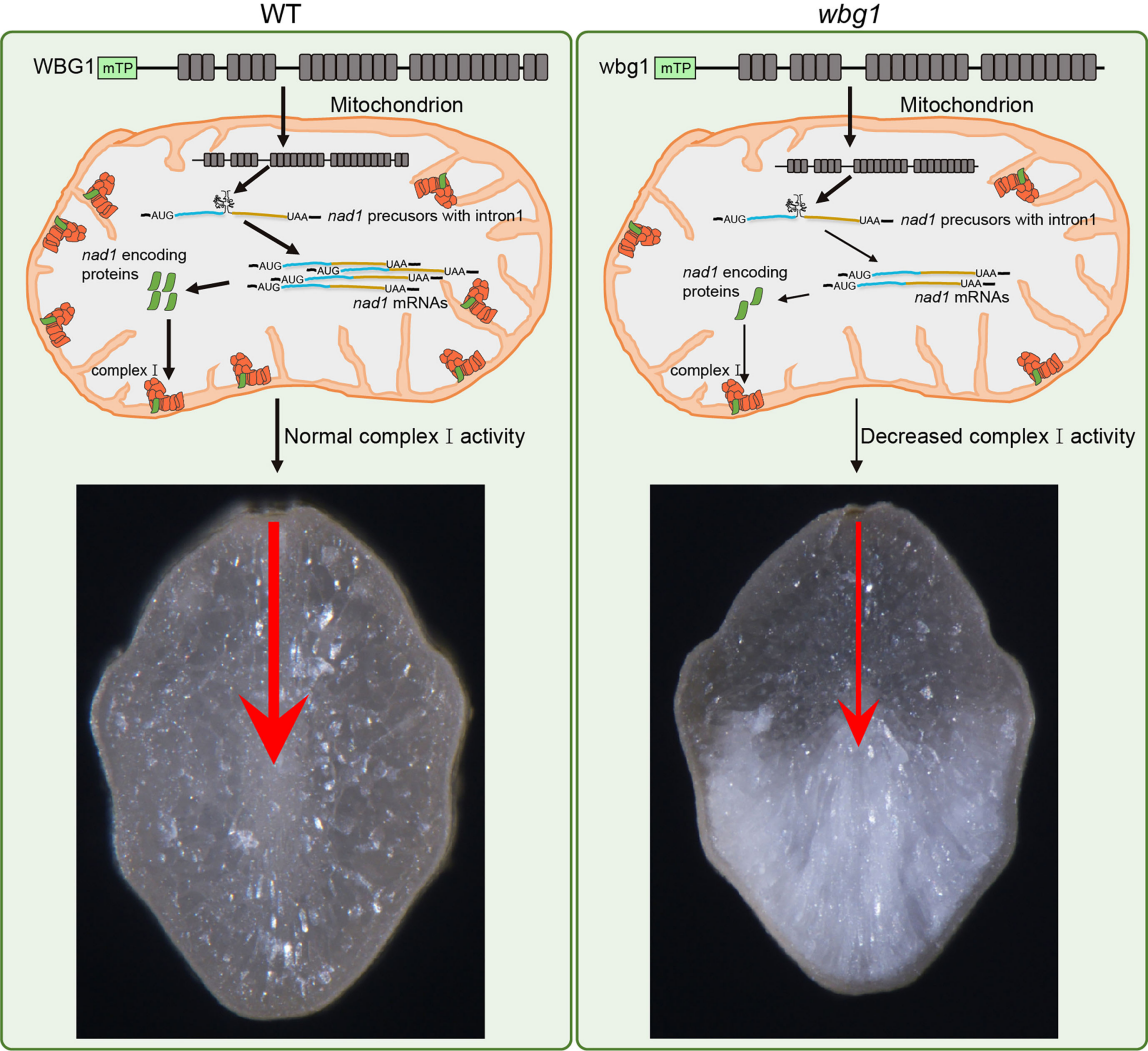
A proposed working model of WBG1 in regulating rice grain chalkiness. In wild-type (WT) (left), *nad1* precursors with intron1 in mitochondria can be normally spliced by WBG1 and produce a certain level of *nad1* mRNA. The *nad1* mRNA encoded protein forms the required amount of mitochondrial complex I, thus maintaining normal mitochondrial function and keeping the transport efficiency of filling assimilates from grain dorsal to belly. mTP, predicted mitochondrion-targeted peptide. Each gray box represents a PPR motif. The blue bold line represents *nad1* exon 1, the brown bold line represents *nad1* exons 2–5, and the curved line between them represents *nad1* intron1. In *wbg1* mutant (right), the loss of two PPR motifs at the C-terminal of wbg1 leads to a reduction in splicing efficiency of *nad1* intron1, resulting in a decrease in *nad1* mRNA level. Then, the activity of complex I, which is composed of *nad1* encoded protein, was decreased. Therefore, the transport efficiency of filling assimilates from the dorsal to belly is reduced, resulting in white belly grain.

Grain filling involves the transport of assimilates from the dorsal portion to the belly portion of grains. Comprehensive analyses, including ^14^C-labeled assimilate analyses, showed that sucrose moves *via* the symplastic pathway in the nucellar epidermis after unloading from the dorsal phloem and is taken up *via* the apoplastic pathway by aleurone cells into the endosperm (Oparka and Gates, 1981a; Oparka and Gates, 1981b; Yang et al., 2018). Previous studies have shown that the mutation in *FLO10* leads to an abnormal aleurone layer with defective mitochondrial function, which may affect the transportation of filling assimilates into the developing endosperm, specifically leading to slow grain filling and a floury endosperm (Wu et al., 2019). In *wbg1*, we found that the activity of respiratory chain complex I and ATP content in the developing endosperm was reduced compared with the wild type, but not to the same extent as in the *flo10* mutant (**Figure 6**). This indicates that mitochondrial activity depends on the level of mature *nad1* mRNA, which is determined by the splicing efficiency of *nad1* intron 1. Furthermore, the white belly phenotype in *wbg1* was distinct from the floury phenotype in *flo10* (**Figure 1A**), indicating that the WBG1 function may be associated with the transverse transportation of filling assimilates in the grain. The loss of the last two PPR motifs in *wbg1* may lead to a reduction in the function of the aleurone layer, which contains a large number of mitochondria. This may result in further reductions in the transport efficiency of filling assimilates from the dorsal to the belly portion of the endosperm, leading to a lack of filling assimilates and resultant chalkiness in the belly. Based on these data, we propose a model for WBG1 regulation of grain chalkiness in rice (**Figure 8**). Further research could usefully focus on improving the efficiency of filling assimilate transport by increasing mitochondrial activity, which should decrease grain chalkiness and enable the cultivation of high-quality rice varieties to meet the market demand.

Previous studies have shown that increased grain width results in grain belly chalkiness (Shi et al., 2019). In this study, we showed that *WBG1* regulates grain filling: a weak mutation in *WBG1* decreases the grain filling rate, leading to the formation of a white belly (**Figures 1A, C**). An analysis of the association between *WBG1* encoding region haplotypes and grain width found clear haplotype differentiation between *indica* and *japonica* rice varieties, which was associated with grain width (**Figure 7**). These results indicate that the *WBG1* haplotype may have an important role in grain width differentiation between *indica* and *japonica* rice, and *WBG1* may have a function in the coordination of rice grain filling rate and grain width.

## Data availability statement

The original contributions presented in the study are included in the article/**Supplementary Material**. Further inquiries can be directed to the corresponding authors.

## Author contributions

MW, SY, and XZ planned and designed the experiments. MC and MW mapped the *WBG1*. MW, MC, RZ, and JY performed all other experiments. SY, GZ, and FY analyzed the data. MW and MC drafted the manuscript. All authors contributed to the article and approved the submitted version.
