# Supplementary material for: A mitochondrion-associated PPR protein, WBG1, regulates grain chalkiness in rice
Source: Front Plant Sci. 2023 Mar 9;14:1136849. doi: 10.3389/fpls.2023.1136849 (PMC10033517; doi:10.3389/fpls.2023.1136849)
Supplement: Supplementary file 1 [file Table_1.docx]

| **Supplemental Table 1** Primers used in this study. | | |
| --- | --- | --- |
| **Use** | **Primer name** | **Sequence (5’ to 3’)** |
| Fine mapping | I3-7-F | CTGCACCGGAGAAATTTGAT |
|  | I3-7-R | CGCATGCAGATGAATAGGTG |
|  | I3-9-F | CAGGCCGGATCTAGTTGAAA |
|  | I3-9-R | CAAAGTGAACAGGCTCGAGAT |
|  | N3-11-F | AAAGTGTTGGTGAGCATAGC |
|  | N3-11-R | TTTGTGTTTGGAGAGACGAG |
|  | W2-F | CTCAAACGGTGGTTCAGA |
|  | W2-R | ACCCAGGAATTTGCTATC |
|  | W5-F | TCCCATCGCCTCGCTTGT |
|  | W5-F | TCACCTCACCACTCCGCTCC |
|  | W8-F | TCTGGAGGTAGGTTTGCTGC |
|  | W8-R | GAAAGAAAGCCCGAAGACTAAA |
|  | W26-F | ATGCCATGCCGCACTT |
|  | W26-R | CCAGCCCAAGCAAACAT |
|  | W35-F | CCGTGTCCACGTTCCACTCTT |
|  | W35-R | GCAAGCTGCCATTTCTACGTTCT |
| Binary vector construction | 1390+pro&CDS-F1 | CGGCGCGCCAAGCTTCAAAATAGTTTCCCGTTTCTGA |
|  | 1390+pro&CDS-R1 | CTTCCACATGGTACCCTAATCCGATCAAAGCTCCTGTCAC |
|  | 1390+pro&CDS-F2 | TCGGATTAGGGTACCATGTGGAAGACTTTGCAGTTATGCA |
|  | 1390+pro&CDS-R2 | AGCGTTAACACTAGTTCACGGGGAGTTCACTTGAGTTGAA |
| Vector detection | F | AATTCGATCAGGCACTTGATTTTC |
|  | R | TCGAGCTGGTCACCTGTAATTCACA |
| qRT-PCR measuring the splicing efficiency of *nad1* intron1 | F | TGCCATATCTTCGCTAGGTG |
|  | R | CAATAGAGACTTCATAAGAGA |
|  | R1 | TTTATGAGCCCTAGCCCTGT |
| Internal control | *Actin*-F | ACCTTCAACACCCCTGCTATG |
|  | *Actin*-R | GCAATGCCAGGGAACATAGTG |
|  | *Ubiquitin*-F | ACCACTTCGACCGCCACTACT |
|  | *Ubiquitin*-R | ACGCCTAAGCCTGCTGGTT |
